# Dental Caries Prevention Knowledge, Attitudes, and Practice among Patients at a University Hospital in Guangzhou, China

**DOI:** 10.3390/medicina59091559

**Published:** 2023-08-28

**Authors:** Rui Jiang, Jiali Yu, Rafiqul Islam, Xiang Li, Ermin Nie

**Affiliations:** 1 Department of Stomatology, First Affiliated Hospital, Sun Yat-Sen University, 58 Zhong Shan Road 2nd, Guangzhou 510080, China; jiangr9@mail.sysu.edu.cn (R.J.); yujli7@mail.sysu.edu.cn (J.Y.); 2 Department of Restorative Dentistry, Faculty of Dental Medicine, Hokkaido University, Kita 13, Nishi 7, Kita-ku, Sapporo 060-8586, Japan; rony12cdc@den.hokudai.ac.jp

**Keywords:** dental caries, oral hygiene, fluoride, fissure sealants, preventive dental knowledge

## Abstract

*Background and Objectives*: This study aimed to assess the knowledge, attitudes, and practice (KAP) of patients regarding dental caries prevention in a university hospital in Guangzhou, China. *Materials and Methods:* A hospital-based KAP survey was conducted in a cross-sectional manner at the First Affiliated Hospital in Guangzhou, China, from 1 September to 30 September 2022. Questionnaires were distributed to eligible patients, resulting in the participation of **251 individuals**. The questionnaire consisted of five sections capturing socio-demographic data and exploring participants’ knowledge, attitudes, practice, and accurate preventive dental caries knowledge. Descriptive statistics and a generalized linear model with univariate tests were used for analysis. *Results***:** The study results show that the knowledge score 7.97 (±0.91) and attitudes score 7.67 (±0.89) among the participants were good while the practice score was 6.80 (±0.81) moderate. The majority of participants identified tooth infections (81.3%), bacteria (92%), and sugar (89.2%) as the main causes of gum bleeding and tooth decay. Brushing (96%) and fluoride (80.5%) were also recognized as essential for preventing tooth decay. Of oral diseases, 94% were recognized as potentially affecting overall health. The vast majority ranging from 92.8% to 98%, believed that oral health is crucial to overall health and that regular check-ups and proper brushing habits are beneficial. There is a significant association of gender with age (*p* = 0.018), occupation (*p* = 0.050), lifestyle habit (*p* = 0.012), and knowledge score; whole education is significantly associated with practice score (*p* = 0.050). *Conclusions:* The majority of patients exhibited accurate knowledge and attitudes with moderate practice towards dental caries prevention, with socio-demographic factors playing a major role. However, effective implementation of dental health education programs across the healthcare system is still required to further enhance outcomes.

## 1. Introduction

One of the most significant oral health issues worldwide is dental caries, which result from the interaction between bacteria and fermentable carbohydrates and can lead to the destruction of the hard parts of teeth [1]. Despite being preventable, dental caries remain prevalent, affecting approximately half of the world’s population, or 3.5 billion people, with 40% of cases going untreated [2]. Unfortunately, dental caries has become a serious global public health concern, with a high incidence of cavities among both children (60–90%) and adults (100%), frequently causing discomfort and pain [3].

There has been a significant increase in patient awareness and education regarding oral health, resulting in a notable reduction in the prevalence of dental caries in most developed countries [4]. The first step towards reducing caries prevalence is understanding preventive dentistry [4]. Studies have shown that parents’ awareness and understanding of dental preventative measures can positively impact the oral health status of their young children [5,6]. These measures include maintaining proper oral hygiene, consuming a healthy and well-balanced diet, and regularly visiting a dental clinic to receive topical fluoride and pit and fissure sealant applications [7].

Several factors have contributed to the increase in dental caries, including the rise in the consumption of sugary foods, poor oral hygiene practices, inadequate teeth brushing habits, hypolasia, and low levels of knowledge [8,9]. Other influential factors include lifestyle, eating habits, and socio-demographic factors [8,9,10]. Dental caries disparities can be partially explained by individuals’ socioeconomic and immigration status [1]. Higher rates of dental caries and their consequences have been linked to lower socioeconomic status and access to quality dental care [3]. Furthermore, the risk and impact of dental caries among affected individuals are exacerbated by insufficient preventive measures like regular dental check-ups and oral hygiene practices, as well as unhealthy dietary changes [1,5,7]. To prevent dental caries effectively, reducing sugar consumption, brushing teeth correctly after meals, and receiving regular check-ups are recommended [11].

In the context of dental caries prevention, it is crucial to examine the specific knowledge, attitudes, and behaviors of individuals towards oral health [12]. Such information is helpful for understanding what should be taught and which behavioral changes are necessary for the improvement of oral health and for developing effective strategies in educating children about good oral health habits [13]. While previous studies conducted in China have explored oral health habits, knowledge, and attitudes of the adult population in urban areas of certain provinces, there is a notable gap in the existing literature concerning a strong theoretical framework that explicitly links knowledge, attitude, and behavior to dental caries prevention practices [14,15]. Consequently, the present study aims to address this limitation by incorporating a theoretical framework to explore the knowledge, attitudes, behavior, and dental caries prevention practices among patients. By doing so, it seeks to provide valuable insights into effective strategies for promoting oral health and preventing dental caries.

China has the world’s largest population, comprising 20% of the total global population [16]. However, as a developing country, China has a relatively limited number of dental care facilities, and access to health insurance is not widely available throughout the country [17]. To assess the prevalence and frequency of dental caries, four national oral health epidemiological surveys have been conducted in China at regular intervals (in 1983, 1995, 2005, and 2015). The reports indicate that 98.4% of the elderly Chinese population has experienced dental caries [18], with more than 60% of cases occurring in 20% of individuals at high risk of developing the disease [19]. These findings highlight the urgent need for the prevention and timely management of dental caries among Chinese citizens. As dental caries continues to become increasingly prevalent in the Chinese population due to inadequate utilization of preventive dental treatments, further research is necessary. This study aims to evaluate the current level of preventive dental knowledge, attitude, and behavior regarding dental caries among patients residing in Guangzhou. The hypothesis of this study is that there is a relationship between the level of accurate preventive dental knowledge and the socio-demographic status among patients residing in Guangzhou. By examining this relationship, the study seeks to provide valuable insights into effective strategies for promoting oral health and preventing dental caries.

## 2. Methods

### 2.1. Ethical Approval

The First Affiliated Hospital of Sun Yat-sen University in Guangzhou, China, provided ethical approval (approval number 2022-424 and date of approval 29 August 2022) for this cross-sectional study.

### 2.2. Study Questionnaire

#### 2.2.1. Questionnaire

The validated questionnaire was adapted from previous cross-cultural studies [20,21]. Five sections of the questionnaire examined participants’ caries prevention knowledge, attitudes, and practice. Knowledge, attitudes, practice, and dental caries prevention knowledge were covered. The questionnaire was adopted, modified, and translated in seven steps: “1. Preparation of a preliminary version; 2. Evaluation and revision; 3. Pretest (verification of item clarity by the target population); 4. Concurrent and content validity; 5. Reliability; 6. Construct validity; 7. Concurrent, construct, reliability, and responsiveness to questionnaire design changes”.

#### 2.2.2. Preparation

A professional translator with a dental specialist translated the English KAPQ into Chinese to create the first draft. The questionnaire was reviewed by dental experts fluent in Mandarin Chinese to ensure that all questions used appropriate terminology. As a final step, a professional translator conducted a back translation to determine where the two translations diverged from one another in the original language.

#### 2.2.3. Evaluation and Modification of Content

A total of four researchers developed the initial survey, which was then reviewed by two bilingual public dental health specialists and a psychometrician. Two stages of testing were used to establish the reliability of the material. To ensure consistency in meaning, the committee compared the original and back-translated English versions of each item. Second, they made sure there was no technical jargon in the finalized Chinese version of the questionnaire by reading it carefully.

#### 2.2.4. Pretest of the Questionnaire

Twenty-five Mandarin Chinese patients were asked to review the questionnaire before it was distributed to the general public to ensure that all of the questions could be answered without ambiguity and that the language was one they were comfortable with. This was accomplished through the use of a randomized interview sample. It was determined that all questions were understandable, and it seems that patients had no problems with comprehension. There have been no changes made to the original.

#### 2.2.5. Validation

We discovered the questionnaire’s factorial structure through confirmatory factor analyses. The internal consistency for KAPQ is quite high (T0 = 0.87, and T1 = 0.91), demonstrating its reliability.

### 2.3. Population

A hospital-based cross-sectional study was carried out at the First Affiliated Hospital between 1 September and 30 September 2022. The source population was all patients who attended the First Affiliated Hospital, San Yet San University. Patients selected from the age of 18 to 65 years old received services from the hospital.

### 2.4. Sample Size Calculation

Sample size was determined for the study using the single population proportion formula, with the following assumptions:

$$n=\frac{Z^{2}p\left(1-p\right)}{d^{2}}.$$

The proportion (***p***) of patients’ response rate was estimated to be 80% based on a previous study [18], with a 95% confidence level (CI), a marginal error (***d***), and a 5% non-response rate. The calculated minimum sample size was 246 participants, but to account for potential missing data, the sample size was increased by 5% to 258 participants.

### 2.5. Inclusion Criteria

Patients were required to meet inclusion criteria, including voluntary participation, regular dental hospital treatment, and informed consent. Patients were required to indicate their willingness and provide written consent to participate in the study.

### 2.6. Exclusion Criteria

To ensure participant selection and study integrity, exclusion criteria were applied. Patients under 18 years old, unable to comprehend the study’s purpose, participants involved in other research or clinical trials, severe systemic diseases, and pregnant females were excluded due to potential medication influence on dental health and treatment outcomes.

### 2.7. Data Collection

The participants in the study were provided with an introductory statement that outlined the purpose of the research and were subsequently informed about the confidentiality measures that would be taken to safeguard their responses. To assess accurate knowledge regarding preventive dental care, participants were asked a series of questions (see supplementary Table S1) specifically related to dental caries prevention. Participants were instructed to provide a response to each question by selecting either ‘Yes’ or ‘No’. The accuracy of participants’ knowledge was determined based on the correctness of their responses to these questions. For instance, if a participant responded ‘Yes’ to the question ‘Are you aware that dental caries can be prevented?’ their response was considered accurate. For each correct response, a score of 1, and for each incorrect response, a score of 0 was assigned to each question in each section. Finally, we calculated the overall score for knowledge, attitudes, practice, and prevention knowledge. We considered 7.1–10.0% to be excellent, 4.1–7.0% to be average, and 0.0–4.0% to be unacceptable.

### 2.8. Statistical Analysis

Statistical analysis of the data was conducted using SPSS software version 22. Responses to the socio-demographic characteristics, knowledge, attitude, and practice sections of the questionnaire were analyzed using a frequency distribution analysis. The mean and standard deviation of the knowledge, attitude, and practice scores were calculated using descriptive analysis. A generalized linear model with univariate analysis was used to examine the factors related to the relationship between socio-demographic characteristics and knowledge, attitudes, and practice. A level of significance at *p* < 0.05 was accepted.

## 3. Results

### 3.1. Socio-Demographic Characteristics

In this study, 251 patients who fulfilled the inclusion criteria voluntarily participated. Among the respondents, 106 (42.2%) were male, and 145 (57.8%) were female. The majority of the patients (32.3%) belonged to the 18–25 age group. Furthermore, almost 80.5% of patients had obtained a college education, and 62.9% were employed. Lifestyle and dietary habits were observed to be mostly consisting of the consumption of sweet food (34.7%), as well as smoking and drinking alcohol (25.1%). The marital status of the participants was found to be 55.8% married and 44.2% unmarried. The socio-demographic characteristics of the participants are provided in Table 1.

### 3.2. Knowledge

In the survey, most of the patients had a good level of knowledge regarding preventive measures against dental caries. Most respondents believed that tooth infections (81.3%), bacteria (92%), and sugar (89.2) could cause gum bleeding and tooth decay. They also found brushing (96%) and fluoride (80.5%) useful in preventing tooth decay and protecting teeth. Additionally, respondents believed that oral diseases (94%) could impact overall health. The majority learned about oral health from their dentist or the internet. The details of the results related to the knowledge regarding preventive dental caries were tabulated in Table 2.

### 3.3. Attitude

The results show that most of the respondents have a good level of positive attitudes regarding preventive measures against dental caries. The survey indicates that a high percentage of respondents, ranging from 92.8% to 98.0%, believe that oral health is essential to good overall health. They also believe that regular check-ups, maintaining oral health, and proper brushing habits are beneficial to their health. In particular, improper brushing habits were associated with gum disease and tooth decay. The majority (93.6%) believed that brushing teeth twice a day improves oral hygiene. The details of the results related to the attitude regarding preventive dental caries were tabulated in Table 3.

### 3.4. Practice

The majority of the participants brush their teeth twice a day (71.7%), followed by those who brush their teeth once a day (15.1%). The majority of participants used toothpaste with fluoride (58.2%), while a smaller percentage reported not using fluoride toothpaste (10.8%), and an unknown number (31.1%) were unsure if their toothpaste contained fluoride or not. Most participants replace their toothbrushes every 3 months (60.6%). The majority of participants (94.0%) give importance to their teeth as much as any other part of their body, while 59.0% reported having routine dental check-ups. The details of the results related to the practice regarding preventive dental caries are tabulated in Table 4.

### 3.5. Accurate Preventive Dental Caries Knowledge

The majority of respondents (87.3%) knew that caries could be prevented, and 91.2% recognized the link between oral hygiene and dental caries. Flossing was recognized as a preventive measure by 74.9% of patients, while 85.7% knew that frequent sugar consumption is associated with dental caries. Topical fluoride applications were acknowledged as a preventive measure by 70.1%, and fissure sealants by 63.3% of patients. Brushing twice a day was recognized as a preventive measure by 74.1% of patients, while only 48.2% knew that preventive dental measures should be taken every six months. Table 5 presents the prevalence of correct knowledge related to preventive dental caries among patients.

### 3.6. Factor Associated between Socio-Demographic Characteristics and Preventive Knowledge

**Figure 1:**
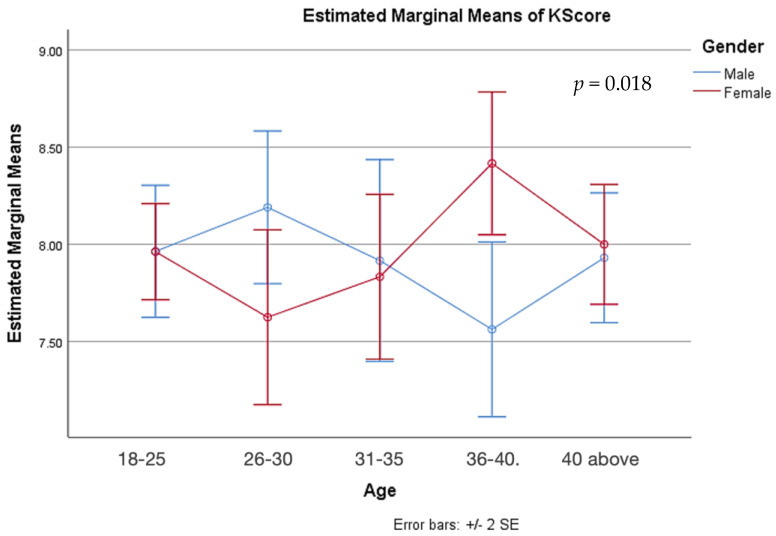
Preventive knowledge between genders among different age groups.

**Figure 2:**
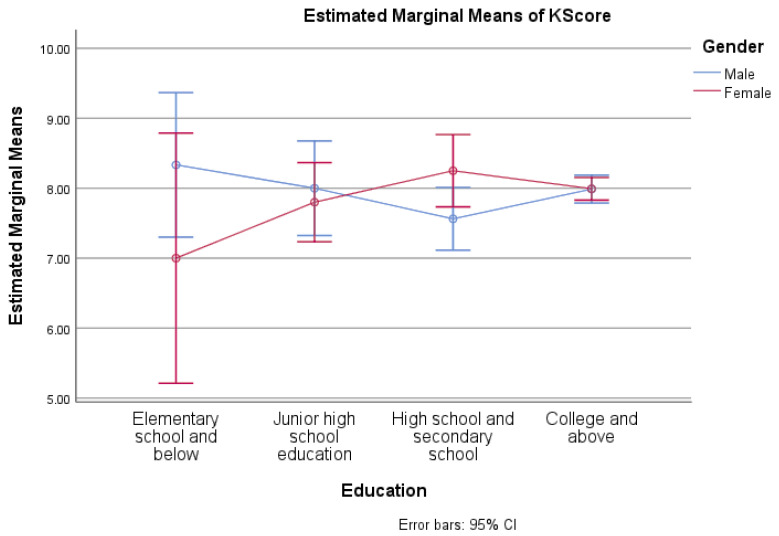
Preventive knowledge between genders with their education level.

**Figure 3:**
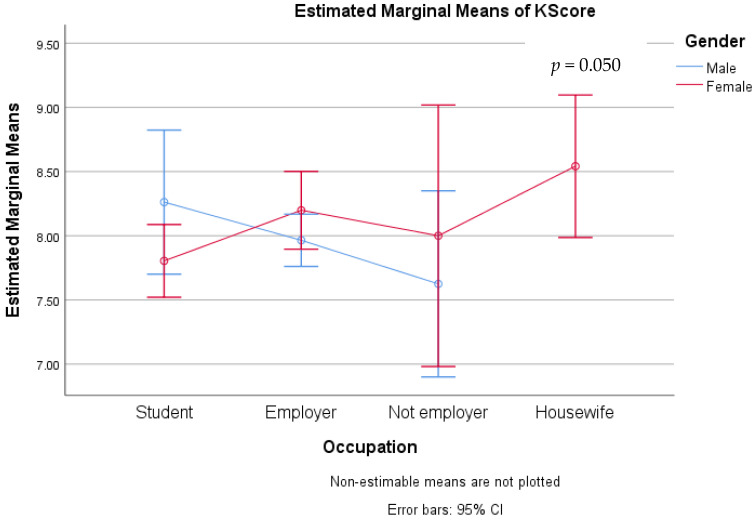
Preventive knowledge between genders with their occupation.

**Figure 4:**
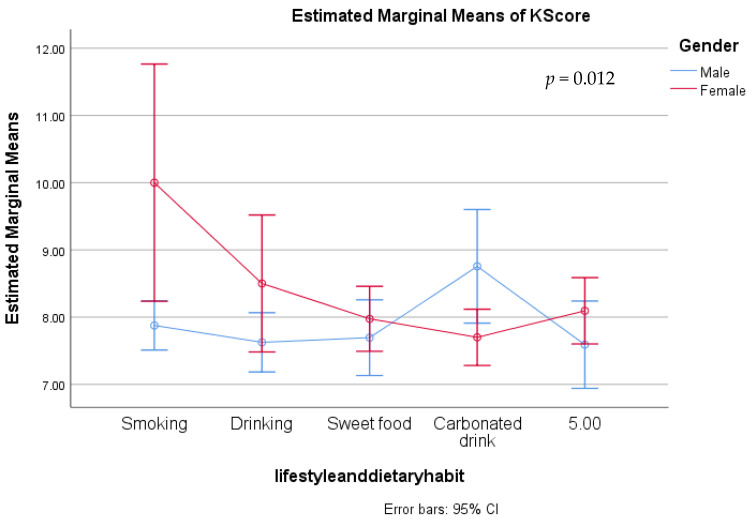
Preventive knowledge between genders with their lifestyle plans and dietary habits.

Using the GLM model and univariate analysis, the factors associated with characteristics and knowledge were evaluated. Individual variables were found to be unrelated to knowledge scores (Table 6). Gender had a significant association with knowledge scores across age groups (*p* = 0.018) (Figure 1), whereas education had no effect on knowledge scores between the sexes (Figure 2). Results also indicated that occupation (*p* = 0.050), lifestyle plans, and dietary habits (*p* = 0.012) were also associated with the difference between male and female knowledge scores (Figure 3 and Figure 4).

### 3.7. Factor Associated between Socio-Demographic Characteristics and Preventive Attitudes and Practice against Dental Caries

The factors associated with responded attitudes and their socio-demographic characteristics are displayed in Table 7. No individual factor is associated with their dental caries prevention attitudes. Table 8 displays the preventative measures associated with socioeconomic factors. Good preventive practice is significantly associated with education level (*p* = 0.050).

## 4. Discussion

In the majority of developed nations, the incidence of dental caries has decreased over the past two decades. However, the prevalence of dental caries has steadily increased in many developing nations in recent years [16]. This increase is largely attributable to increased sugar consumption and inadequate fluoride exposure [22]. In addition, the use of biomimetic hydroxyapatite as a substitute for fluoride has been investigated, yielding valuable insights into its potential to reduce the incidence of dental caries and lesions [23,24]. Despite being the world’s most populous and most rapidly developing country, China’s various regions exhibit notable differences in economic status, culture, education, and diet. Consequently, the prevalence of oral diseases varies significantly by region [25].

Several statistically significant associations between dental caries prevention and socioeconomic factors were found, suggesting that the hypothesis is correct. The majority of survey participants, as shown by the present study’s findings, blamed tooth infections, bacteria, and sugar for gum bleeding and tooth decay. This result agrees with previous research [16,26] that stressed the significance of good oral hygiene and the consequences of neglecting one’s oral health. In addition, those who participated understood the role that brushing and fluoride play in warding off cavities and safeguarding teeth. This is consistent with what the American Dental Association advises [27], which is to brush your teeth twice a day with fluoride toothpaste. The fact that respondents thought oral diseases could affect general health is significant because it demonstrates the connection between oral health and overall health. Several systemic diseases, such as cardiovascular disease and diabetes, have been linked to poor oral health, and studies support this finding [28,29]. Finally, most respondents said they found out about the importance of good oral hygiene from either their dentist or the Internet. This finding highlights the need for trustworthy online resources and the role of dental professionals in public education about oral health.

The findings of our study showed that the majority of patients had a good understanding of the importance of oral hygiene and dental caries prevention. This is encouraging, as education plays a crucial role in promoting good oral health practices. However, despite this knowledge, the prevalence of dental caries in China is still on the rise, as several studies have reported [30,31,32]. According to a study by Liu et al. (2019), caries prevalence in Northeast China was as high as 84% among children ages 6–12 and 76.92% among those ages 13–20 [33]. These alarming statistics indicate that despite people being aware of the importance of good oral health practices, they are still struggling to maintain them. Several other studies have also highlighted the high risk of dental caries among the Chinese population [34,35,36]. The reasons for this could be multifactorial, including diet, lack of access to dental care, and poor oral hygiene practices [37]. However, addressing these issues is essential for stopping the spread of dental caries. Education about dental hygiene and caries prevention is important, but it is not sufficient on its own. Dentists can play a pivotal role in this by helping patients change their mindsets about the importance of dental health. They might suggest going to the dentist regularly, promoting nutritious eating, and stressing the significance of good oral hygiene [38].

Our study results revealed that nearly 74.9% of patients believed that flossing could help prevent dental caries. However, the practice of flossing is strongly influenced by an individual’s lifestyle, which is, in turn, influenced by factors such as socioeconomic status, level of education, and other habits [39]. These findings suggest that in addition to educating patients about flossing, efforts should also be made to address the underlying lifestyle factors that impact their oral health practices [40]. In contrast, most of the patients in our study were aware that sugar consumption could lead to dental caries. However, many believed that simply brushing their teeth after consuming sugary foods and drinks before bedtime was sufficient to protect their teeth [41]. This highlights the need to educate patients on the importance of minimizing sugar consumption and adopting other preventive measures, such as regular dental check-ups and good oral hygiene practices. More than half of the patients also recognized the preventive benefits of topical fluoride application and fissure sealants. This is likely due to the regular visits to dental clinics or hospitals where they learned about these procedures from their dentists. Interestingly, studies have shown that fissure sealants can reduce the risk of dental caries by up to 37%, indicating their effectiveness in preventing caries [40,42].

In our study, most patients reported that they should visit the dental clinic or hospital every six months for a regular check-up, although many were unsure about the appropriate timing for such visits [4]. It was also found that patients often only seek dental care when they experience pain or discomfort with their teeth [43]. These findings emphasize the need for patient education on the importance of regular dental check-ups for preventive care and early detection of oral health issues. On a positive note, the majority of patients in our study reported using fluoridated toothpaste and brushing their teeth twice a day. They were also aware of the benefits of fluoride for dental health. This may be attributed to regular dental visits where patients receive guidance from their dentists on good oral hygiene practices, including the use of fluoridated toothpaste [44,45]. Alternatively, it could be due to patients’ own knowledge and understanding of the advantages of fluoride for maintaining good oral health [44]. Overall, these findings highlight the importance of patient education on the appropriate timing of dental visits, as well as the benefits of good oral hygiene practices and preventive care measures such as the use of fluoridated toothpaste. By improving patient knowledge and understanding of these key factors, we can work towards reducing the incidence of dental problems and promoting better oral health outcomes.

Regular dental check-ups are undoubtedly important for maintaining good oral health and preventing dental caries [15]. However, it is equally crucial for individuals to take responsibility for their own oral care at home as a preventive measure against oral diseases such as caries [46]. Dentists play an important part in this context by educating and assisting patients in achieving good oral health. Dentists have a responsibility to educate their patients about the importance of maintaining good oral hygiene, including the best ways to brush and floss, the connection between diet and dental health, and the negative effects of sugar consumption. Oral health professionals have a responsibility to inform patients about the range of options available for preventative care and treatment, including fluoride toothpaste. Dentists can better equip their patients to prevent and manage dental caries if they educate them on how to do so on their own.

There were a number of caveats to this research. We were unable to include more medical facilities due to time and resource constraints. Therefore, the findings of this study cannot be extrapolated to the entire state. In addition, we only included one major hospital in this province, which does not accurately portray the wide range of practices and perspectives present throughout the country’s healthcare system. We suggested conducting a national study with multiple study sites in each province to obtain a complete picture of people’s preventative dental caries-related knowledge, attitudes, and practice.

## 5. Conclusions

The results of this study show that patients generally have sound knowledge of measures to prevent dental caries, with demographic factors playing a major role. However, dental health education programs still need to be effectively implemented across all areas of China’s healthcare system. Fortunately, the prevalence of dental caries can be reduced by increasing public awareness of preventive dentistry and encouraging the use of available preventive measures.

## Figures and Tables

**Table 1:** The socio-demographic characteristics of the participants.

Socio-Demographic Characteristics		Count/% n = 251
Age (years)	18–25	81/32.3
	26–30	37/14.7
	31–35	30/12.0
	36–40	40/15.9
	40 above	63/25.1
Gender	Male	106/42.2
	Female	145/57.8
Education	Elementary school and below	4/1.6
	Junior high school	17/6.8
	High and secondary school	28/11.2
	College and above	202/80.5
Occupation	Student	61/24.3
	Employer	158/62.9
	Unemployed	12/4.8
	Housewife	20/8.0
Income	Less than 3000	48/19.1
	3000–5000	47/18.7
	More than 5000	156/62.2
Lifestyle and dietary habits	Smoking	36/14.3
	Drinking alcohol	20/8.0
	Sweet food	87/34.7
	Carbonated drink	45/17.9
	Both smoking and drinking alcohol	63/25.1
Marital status	Married	140/55.8
	Single	111/44.2

**Table 2:** The level of knowledge of patients regarding preventative measures against dental caries.

Knowledge	Frequency (%)	
Knowledge score	7.97 (±0.91) *	
A tooth infection causes gum bleeding?	204 (81.3)	
Is it normal for gums to bleed when brushing?	53 (21.1)	
Bacteria can cause inflammation of the gums?	231 (92.0)	
Bacteria can cause tooth decay?	224 (89.2)	
Eating sugar cause tooth decay?	224 (89.2)	
Brushing is useful in preventing bleeding gums?	194 (77.3)	
Brushing is useful in preventing tooth decay?	241 (96.0)	
Brushing can protect teeth?	240 (95.6)	
Fluoride is useful in protecting teeth?	202 (80.5)	
Oral disease may affect the health of the entire body?	236 (94.0)	
How do you learn about oral health?	from internet	104 (41.4)
	from dentist	130 (51.8)
	from parents	6 (2.4)
	from colleague	11 (4.4)

* Mean (tsandard deviation).

**Table 3:** The level of attitude of patients regarding preventative measures against dental caries.

Attitude	Frequency (%)
Attitude score	7.67 (±0.89) *
Oral health is important to life	243 (96.8)
Regular oral check-ups are essential	237 (94.4)
Maintaining oral health promotes good health	246 (98.0)
Keeping your teeth clean and healthy is beneficial to your health	245 (97.6)
Improper brushing leads to gum disease	233 (92.8)
Improper brushing leads to tooth decay	215 (85.7)
Brushing teeth twice a day improves oral hygiene	235 (93.6)

* Mean (standard deviation).

**Table 4:** The level of practice of patients regarding preventative measures against dental caries.

Practice	Frequency (%)	
Practice score	6.80 (±0.81) *	
Frequency of brushing	3 times a day	30 (12.0)
	2 times a day	180 (71.7)
	Once a day	38 (15.1)
	3–6 times a week	1/(0.4)
	1–2 times a week	2/(0.8)
Toothpaste used	Fluoride	146/(58.2)
	No fluoride	27 (10.8)
	Unknown	78 (31.1)
Frequency of toothbrush replacement	3 months	152 (60.6)
	3–6 months	67 (26.7)
	6–12 months	12 (4.8)
	Replace when broken	20 (8.0)
I give importance to my tooth as much as any part of my body	236 (94.0)	
I do routine dental check-ups	148 (59.0)	

* Mean (standard deviation).

**Table 5:** The prevalence of accurate knowledge related to dental caries prevention among patients.

Preventive Dental Knowledge	Frequency (%)	
Do you know dental caries can be prevented	219/87.3	
Do you know dental caries is related to oral hygiene	229/91.2	
Do you know flossing can prevent dental caries	188/74.9	
Do you know dental caries is related to sugar intake	215/85.7	
Do you know topical fluoride can prevent dental caries	176/70.1	
Do you know fissure sealant can prevent dental caries	159/63.3	
Brushing daily can prevent dental caries	1 time/day	47/18.7
	2 times/day	186/74.1
	3 times/day	18/7.2
Visit dental clinic or hospital regularly as a measure to prevent dental caries	Within 6 months	121/48.2
	Within 1 year	38/15.1
	Within 2 years	34/13.5
	Do not know	58/23.1

**Table 6:** Factor associated between socio-demographic characteristics and preventive knowledge.

Factors	Mean Square	F-Stat	*p*-Value
Age	0.079	0.032 (4)	0.997
Gender	0.157	0.068 (1)	0.807
Education	0.543	0.119 (3)	0.943
Occupation	0.262	0.105 (3)	0.952
Lifestyle and dietary habits	1.187	0.449 (4)	0.771

**Table 7:** Factor associated between socio-demographic characteristics and preventive attitudes.

Factors	Mean Square	F-Stat	*p*-Value
Age	0.655	0.699 (4)	0.632
Gender	3.014	3.257 (1)	0.135
Education	0.325	0.428 (3)	0.733
Occupation	0.663	0.875 (3)	0.455
Lifestyle and dietary habits	1.574	2.076 (4)	0.085

**Table 8:** Factor associated between socio-demographic characteristics and preventive practice.

Factors	Mean Square	F-Stat	*p*-Value
Age	0.102	0.155 (4)	0.951
Gender	0.698	1.063 (1)	0.352
Education	1.136	9.250 (3)	**0.050**
Occupation	0.257	0.289 (3)	0.839
Lifestyle and dietary habits	1.061	0.665 (4)	0.617

## Data Availability

The data presented in this study are available on request from the corresponding author.

## Supplementary Materials

The following supporting information can be downloaded at: https://www.mdpi.com/article/10.3390/medicina59091559/s1, Table S1: Questionnaire of the study.
