# Supplementary material for: Dental Caries Prevention Knowledge, Attitudes, and Practice among Patients at a University Hospital in Guangzhou, China
Source: Medicina (Kaunas). 2023 Aug 28;59(9):1559. doi: 10.3390/medicina59091559 (PMC10535681; doi:10.3390/medicina59091559)
Supplement: Supplementary file 1 [file medicina-59-01559-s001.zip › medicina-2524054-supplementary.pdf]

Supplementary Table S1: Questionnaire of the study.

**Sociodemographic Characteristics**	**Knowledge**	**Attitude**	**Behavior**	**Preventive Dental Knowledge**
Age	Do you think a tooth infection causes gum bleeding?	Do you think oral health is important to life?	How often do you brush your teeth?	Are you aware that dental caries can be prevented?
Gender	Do you think it is normal for gums to bleed when brushing?	Do you think regular oral check-ups are essential?	What types of toothpaste do you use?	Do you know dental caries is related to oral hygiene?
Education	Do you think bacteria can cause inflammation of the gums?	Do you think maintaining oral health promotes good health?	How frequently do you replacement your toothbrush?	Do you know flossing can prevent dental caries?
Occupation	Do you think bacteria can cause tooth decay?	Do you believe that maintaining clean and healthy teeth is beneficial to your overall health?	Do you give importance to your tooth as much as any part of your body?	Do you know dental caries is related to sugar intake?
Income	Do you think eating sugar cause tooth decay?	Do you think improper brushing leads to gum disease?	Do you regularly undergo routine dental checkups?	Do you know topical fluoride can prevent dental caries?
Lifestyle and dietary habit	Do you believe that brushing can prevent bleeding gums?	Do you think improper brushing leads to tooth decay?		Are you aware that fissure sealant can help prevent dental caries?
Marital status	Do you think brushing is useful in preventing tooth decay?	Do you believe that brushing your teeth twice a day is beneficial for oral hygiene?		Do you believe that daily brushing can help prevent dental caries?
	Do you believe that brushing can help protect teeth?			Do you believe that regularly visiting a dental clinic or hospital can help prevent dental caries?
	Do you think fluoride is useful in protecting teeth?			
	Do you consider your teeth to be as important as any other part of your body?			
	How do you learn about oral health?			
